# Dimethyl adipimidate/Thin film Sample processing (DTS); A simple, low-cost, and versatile nucleic acid extraction assay for downstream analysis

**DOI:** 10.1038/srep14127

**Published:** 2015-09-15

**Authors:** Yong Shin, Swee Yin Lim, Tae Yoon Lee, Mi Kyoung Park

**Affiliations:** 1 Institute of Microelectronics, A*STAR (Agency for Science, Technology and Research), 11 Science Park Road, Singapore Science Park II, Singapore 117685.; 2 Asan Institute for Life Science, Department of Convergence Medicine, University of Ulsan College of Medicine, Asan Medical Center, 88 Olympicro-43gil, Songpa-gu, Seoul, Korea.; 3 Department of Technology Education, Chungnam National University, Daejeon 34134, Korea.

## Abstract

Sample processing, especially that involving nucleic acid extraction, is a prerequisite step for the isolation of high quantities of relatively pure DNA for downstream analyses in many life science and biomedical engineering studies. However, existing methods still have major problems, including labor-intensive time-consuming methods and high costs, as well as requirements for a centrifuge and the complex fabrication of filters and membranes. Here, we first report a versatile Dimethyl adipimidate/Thin film based Sample processing (DTS) procedure without the limitations of existing methods. This procedure is useful for the extraction of DNA from a variety of sources, including 6 eukaryotic cells, 6 bacteria cells, and 2 body fluids in a single step. Specifically, the DTS procedure does not require a centrifuge and has improved time efficiency (30 min), affordability, and sensitivity in downstream analysis. We validated the DTS procedure for the extraction of DNA from human body fluids, as well as confirmed that the quality and quantity of the extracted DNA were sufficient to allow robust detection of genetic and epigenetic biomarkers in downstream analysis.

Recent advances utilizing convergence of knowledge (science and engineering) in the field of biological applications have brought about the development of tools for the downstream analysis of genetic and epigenetic variants of genes, such as polymerase chain reaction (PCR), real-time (RT)-PCR, microarray, and next-generation sequencing^1^,^2^,^3^,^4^. Technical advances (which are integral to these methodologies) for sample processing to obtain good quality DNA from various samples, including cells, bacteria, blood, and urine, are desirable. Such advances could allow for the downstream analysis to proceed with improved rapidity, simplicity, and high sensitivity and specificity in a broad range of biological applications, including biomarker detection, molecular identification, and other genomic applications^5^,^6^,^7^,^8^,^9^,^10^,^11^,^12^. However, existing methods have not met the needs in sample processing, which still remains as a bottleneck for downstream biological applications, due to severe constraints in processing.

Conventional nucleic acid extraction methods based on filter/membrane with/without chemical reagents include salting-out, organic (phenol/chloroform), cesium chloride density gradient, anion-exchange and chaotropic/non-chaotropic extractions. These have been commonly utilized in a variety of biological applications, but they usually lack standardization and reproducibility for good yields and quality of DNA when used by different persons or when applied to different samples^13^,^14^,^15^,^16^,^17^,^18^. Despite the hundreds of nucleic acid extractions performed every day in biological and clinical laboratories, the methods used are still tedious, time-consuming, labor-intensive, and require laboratory instruments such as centrifuges. Furthermore, the numerous hand-on time including pipetting steps can lead to error and contamination and can subsequently limit on-site processing abilities^13^,^14^,^15^,^16^,^17^,^18^. In order to overcome these limitations, a myriad of advances based on engineering techniques have been developed in the last few decades; these techniques link nucleic acid to micro/nano scale susbtrates and use silica-based surface affinity, electrostatic interactions, nanoporous membrane filtration, and even functionalized micro/nano-particles in a tiny room to improve extraction^19^,^20^,^21^,^22^,^23^,^24^. These techniques have been successful in providing DNA in small and concentrated volumes by using microdevices that can also facilitate a decrease in the total time of processing and cost by reducing the sample size and reagents consumed. However, they require complicated device fabrication steps including mask, deposition, photolysis, and etching in a clean room; these steps significantly increase the cost of the procedure^25^,^26^,^27^,^28^,^29^,^30^. Given that all techniques depend on the purpose of the application, an ideal method will offer high DNA quality and purity and time-efficiency, with no need for centrifugation; such a method should also have ease of application and should be extremely adaptable for subsequent downstream DNA analysis. Furthermore, application as a medical diagnostic tool requires DNA analysis to be performed on a reasonably short time scale in order to detect disease states that require quick action.

To the best of our knowledge, this is the first report of a single step on chip sample processing procedure with less instrument that leverages the advantages of dimethyl adipimidate (DMA)-nucleic acid binding and a simple thin film preparation scheme, thereby addressing all the above mentioned disadvantages of existing sample processing methods. DMA is a non-chaotropic reagent that has been reported by our group as a DNA capture reagent through the recognition of the amine groups on the sticky ends of the fragmented DNA in a silicon microchannel device^31^,^32^,^33^,^34^. Along with DMA, the thin film device is synthesized by laser fabrication. The use of this device helps remove any complicated steps in the fabrication, such as etching and use of bonding reagents or mass manufacturable molding methods. Our current device offers many advantages over the existing methods including low costs, simple fabrication, disposability, flexibility, and transparency^35^,^36^,^37^. This combination of these two advanced techniques provides a simple to use [i.e., One-shot input of mixed sample and reagents, and reaction steps (such as cell lysis, DNA isolation, and purification) occur simultaneously in a single channel], low-cost, and flexible device for the extraction of DNA for a broad spectrum of biological applications and does not require centrifuges and complicate fabrication steps.

## Results

### Characterization of DTS assay

**Figure 1:**
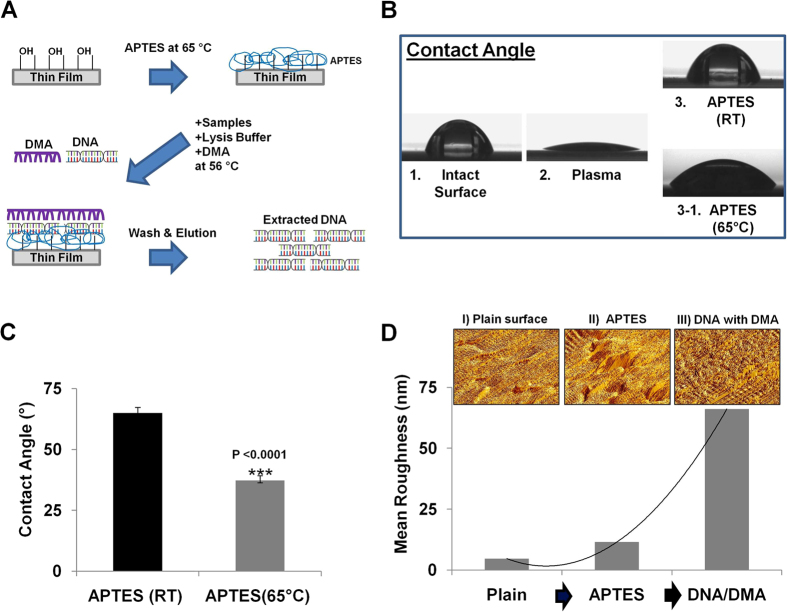
Characterization of DTS assay. (**A**) Schematic process of the DTS assay. Mixture solution including lysis buffer, samples, and DMA is added into the thin film device to extract DNA. The solution is incubated at 56 °C for 20 min to capture DNA through DMA reagent on the surface of the thin film. The DNA is quickly washed and eluted. (**B**) Water contact angle was measured to check the hydrophilicity change of the surface by APTES modification. (**C**) Amine-reactive groups on the surface were well-created by APTES at 65 °C for 20 min. (***) indicated statistically significant by the student *t-test*. All error bars indicate the standard error of the mean based on at least 3 independent experiments. (**D**) AFM 3D image and the surface roughness confirming the functionality of DNA/DMA complex on the surface.

**Figure 2:**
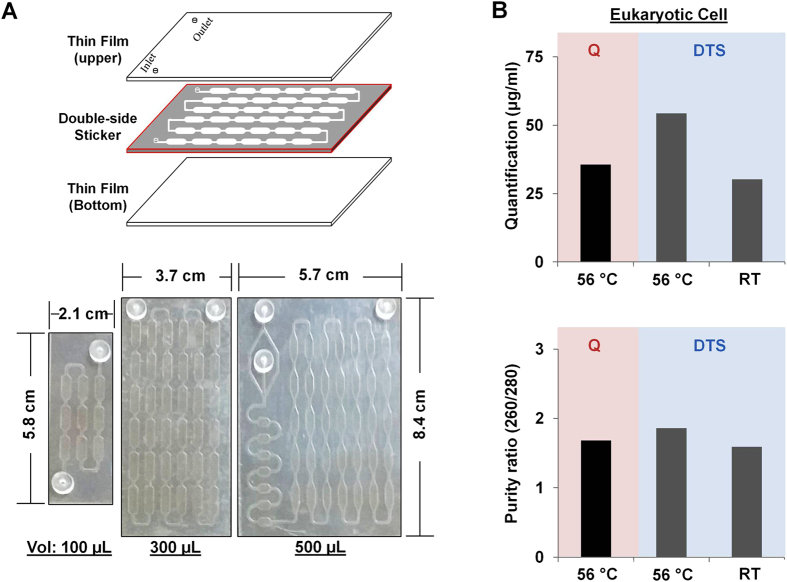
Application of DTS assay in eukaryotic cells. (**A**-upper) DTS device is fabricated using a laser cutting machine with simplicity, rapidity, and low-cost. (**A**-lower) Flexibility of thin film devices. The thin film devices that are fabricated using a laser cutting machine depending on the reaction volume (100, 300, and 500 μL). (**B**) The quantity and purity of the DNA extracted from eukaryotic cells (MCF7, breast cancer cell line) by using the DTS assay (light blue) and Qiagen kit (red) at 56 °C.

In Fig. 1A, the DTS (DMA/Thin film based Sample processing) assay includes three steps; sample lysis/incubation, washing, and elution, that can be performed in a single device without a centrifuge. A mixture solution of sample (eukaryotic cells, bacterial cells (gram^−^ and gram^+^), whole blood, or urine), lysis buffer, and DMA is added by pipetting into the thin film device, which is activated beforehand on the surface with (3-aminopropyltriethoxysilane (APTES), which leads to form amine group for terminal functionalities of the organic molecules. The mixture on the thin film device is then incubated for 20 min at 56 °C to extract DNA from the samples and isolate DNA through the DMA reaction, which uses the cross-linking mechanism between DNA and DMA due to the bi-functional amine reactive group of DMA interacts with the amino groups in DNA. After the incubation, PBS is added for 5 min at room temperature (RT) to wash out the debris. Finally, the elution buffer is added to collect the extracted DNA, which is subsequently used in the downstream analysis of biomarkers (Fig. 1A, Table S1). First, in order to successfully combine the DMA technology with a thin film device for the extraction of DNA, the surface of the thin film needs to be functionalized with APTES, which leads to convert hydrophobic surface to hydrophilic surface by the formation of amine-reactive groups on the surface. This exposed amine-reactive groups on the surface that are critical for binding the DNA present in the sample. Due to the properties of thin films (such as easy deformation from stresses including high-temperature and exposure to solvents)^38^,^39^, we performed functionalization of the thin film at low-temperature and in a water-based solution to attach the amine-reactive groups on the surface. To determine the reliability of the protocol, we measured the hydrophilicity of the surface with a reaction that included combining plasma and APTES, followed by incubation for 10 to 60 min at RT or 65 °C and then washing with water. The hydrophilicity of the surface, was then tested by measuring the water contact angle and was found to be significantly dependent on the incubation time and temperature. The hydrophilicity of the surface is also important for capturing DNA by the DMA reagent that can be rendered by functionalization of APTES. Figure 1B,C and S1A shows that the formation of the amine-reactive group on the surface of the thin film by APTES can be achieved at low temperature (65 °C), is fast (<30 min), and occurs readily in water solution (instead of an organic solvent solution), which prevents surface deformation associated with high temperature (80–120 °C), and long incubation times (>2 hr), which can lead to nonfunctional thin films (Fig. 1 and Fig. S1A). Furthermore, surface roughness of the thin film obtained from atomic force microscope (AFM) was monitored to confirm the functionalization with binding (Fig. 1D). The attachment of DNA/DMA complex on the surface resulted in a steeply changed in the surface roughness (66 nm) compared to both the plain surface (4.5 nm) and surface after APTES modification (11 nm) (Fig. 1D). Next, several types of thin films on double-side tape, which is used to make a simple microfluidic channel for flowing the solution, were cut by using a laser machine and then assembled for use with various volumes of samples (Fig. 2A). The thin film device has no filter/membrane and is combined with DMA for the extraction of DNA directly from raw samples with the lysis buffer solution. DMA has been used as an amino-reactive cross-linking agent for cells, proteins, and nucleic acids by the formation of reversible cross-linking structure because it contains bi-functional imidoesters^31^,^32^,^33^,^34^. In order to check whether DMA can bind DNA from a unpurified samples of cells, proteins, and nucleic acids, we performed a binding affinity assay using silicon microring resonators (SMRs), which is a label-free and real-time detection method based on refractive index change^40^,^41^,^42^. We observed that the kinetics of DMA binding with DNA is relatively rapid compared to that of proteins during short incubation time (20 min) (Fig. S1B). The interaction with protein over short time periods appears to be relatively negligible. Significantly, we show here that the rapid and strong interaction between the DMA and DNA, not protein, would be able to lead to highly efficient DNA extraction from unprocessed samples (Fig. S1B).

### Application and validation of DTS assay for DNA extraction from eukaryotic cells and bacterial cells

In order to test the efficiency of the DTS assay that couples of DMA and a thin film device, we measured the recovery rate of the input DNA (1 μg of human genomic DNA) in the mixture solution including lysis buffer with and without DMA by spectrophotometer. More than 95% of the DNA was recovered with DMA in the mixture and less than 50% of DNA in a mixture solution was recovered without DMA (Fig. S1C). In addition, we used the DTS assay for the direct extraction of DNA from eukaryotic cells (1 × 10^7^ cells of MCF7 breast cancer cells). We compared the DTS assay (<30 min) with a Qiagen extraction kit (>60 min) for standardization and reproducibility of the DNA extracted and confirmed critical parameter measurements such as quantity, purity, sufficient removal of PCR inhibitors, processing time, and cost (Fig. 2, Table 1). All experiments were repeated at least three times. The quantity and purity of the DNA extracted from the DTS assay was comparable to that obtained with the Qiagen kit at 56 °C (Fig. 2CB, Table 1). The DTS assay can be used for the extraction of DNA from eukaryotic cells at both 56 °C and RT, indicating that this would allow use in a setting with limited sources (Fig. 2B). For genetic analysis, we used the DNA extracted from each sample group with both the DTS assay and the Qiagen kit, as target templates (5 μL of each) for the amplification of the *Actin* gene using quantitative real time (qRT)-PCR. We observed that the actin gene was strongly amplified in both Qiagen (ct: 26.56 ± 0.25) and DTS (26.77 ± 0.35) assays. In addition, we examined the integrity of total DNA extracted from DTS device. The total DNAs extracted by using both DTS device and Qiagen kit were shown no any fragmentation of the DNA on 1% agarose gel (Figure S2A). Furthermore, we performed reuse testing in order to verify whether how many times of one device can be used for the nucleic acid extraction. We showed that the DTS device could be re-used at least twice in a single device (Figure S2B). Despite the result of the reuse testing, the DTS device was devised as a disposable chip for nucleic acid extraction due to cross-contamination issue between samples.

**Figure 3:**
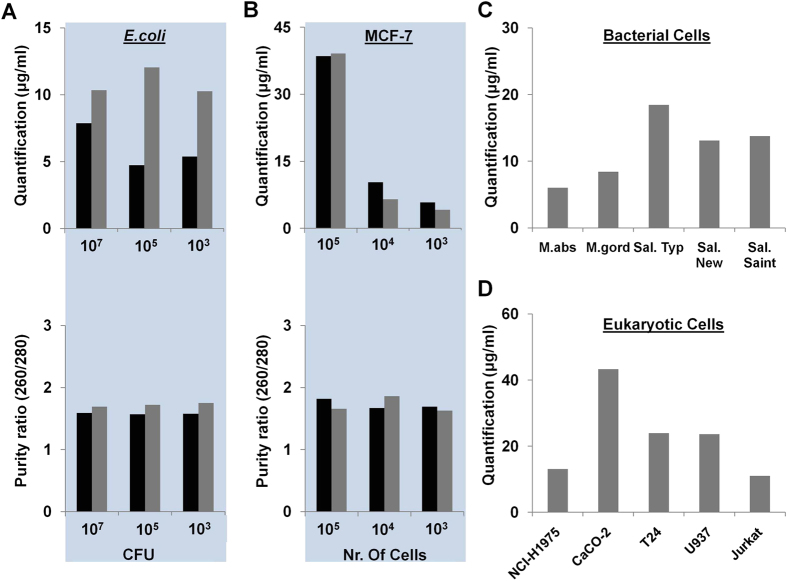
Application of DTS assay in 12 samples including 6 bacterial cells and 6 eukaryotic cells. (**A**) Capacity of the DTS assay with *E. coli* containing range from 1 × 10^3^ to 10^7^ colony forming unit (CFU). The colors represent the type of assay; Black (Qiagen kit), and Grey (DTS). The quantity (upper) and purity (lower) of the DNA extracted from *E. coli* are measured by the spectrophotometer. (**B**) Capacity of the DTS assay with MCF7 cells containing range from 1 × 10^3^ to 10^5^ cells. The colors represent the type of assay; Black (Qiagen kit), and Grey (DTS). The quantity (upper) and purity (lower) of the DNA extracted from MCF7 are measured by the spectrophotometer. (**C**,**D**) Capacity of the DTS assay with (**C**) 10^7^ CFU bacterial cells (mycobacteria and salmonella strains) and (**D**) 10^5^ eukaryotic cells (NCI-H1975: lung, Caco-2: colon, T24: bladder, U937: bone marrow, and Jurkat: lymphocyte). The quantity and purity of the DNA extracted from both eukaryotic and bacterial cells are measured by the spectrophotometer.

**Figure 4:**
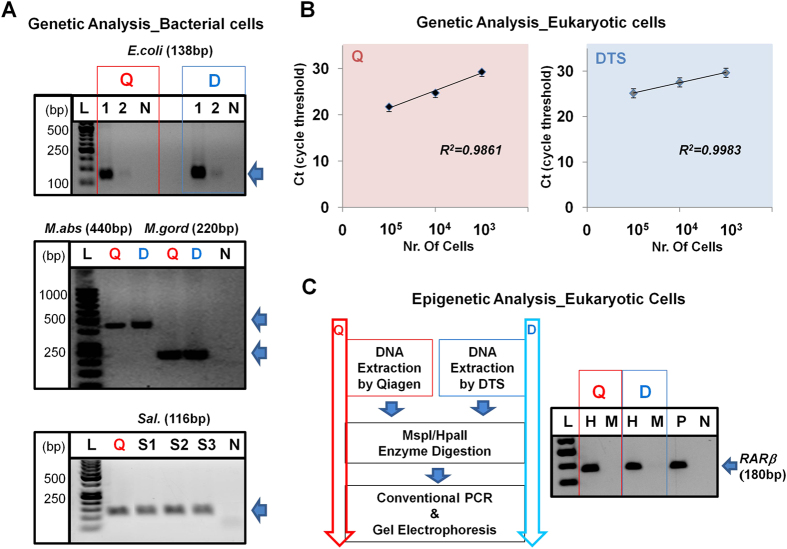
Validation of DTS assay for the downstream analysis in bacterial and eukaryotic cells. (**A**) The downstream analysis in bacterial cells - Genetic analysis with *E. coli, M. abscessus, M. gordonae, and Sal.* strains using conventional PCR with the DNA extracted from either the Qiagen kit (red) or DTS assay (light blue) and then gel electrophoresis analysis after PCR. [1: 10^5^, 2: 10^3^ CFU samples, N: no DNA (negative), S1: *Sal. Typhimurium*, S2: *Sal. Newport*, S3: *Sal. Saintpaul*]. (**B**) The downstream analysis in eukaryotic cells - Genetic analysis with *HRAS* gene using the DNAs extracted from the Qiagen kit (left, red; 10^5^: 21.75 ± 0.10, 10^4^: 24.76 ± 0.25, 10^3^: 29.32 ± 0.12) and the DTS assay (right, light blue; 10^5^: 25.17 ± 0.31, 10^4^: 27.57 ± 0.14, 10^3^: 29.66 ± 0.02). All error bars indicate standard error of the mean based on at least 3 independent experiments. (**C**) The downstream analysis in eukaryotic cells - Epigenetic analysis with *RARβ* gene using the DNAs extracted from the Qiagen kit (left-red) and the DTS assay (right-light blue). The DNA extracted was digested with methylation specific endonucleases (MspI/HpaII) and then gel electrophoresis analysis after conventional PCR. [H: HpaII, M: MspI, P: positive, and N: negative].

Next, to further evaluate the capacity of the DTS assay, we tested bacterial DNA extracted from six different types of bacterial cells including *Escherichia coli*^43^,^44^, *Mycobacterium abscessus, Mycobacterium gordonae*^45^,^46^, and *Salmonella Stranins (Typhimurium, Newport, and Saintpaul)*^47^. The sample solution containing *E. coli* ranging from 1 × 10^3^ to 10^7^ colony forming unit (CFU) was in a same volume of 100 μL. As provided in Fig. 3A (upper and lower), we showed that the quantity and purity of extracted DNA from the DTS assay is similar to that isolated by the Qiagen kit (Table 1). Additionally, we tested the capacity of the DTS assay with other bacterial species including *M. abscessus, M. gordonae*, *Sal. typhimurium, Sal. newport, and Sal. saintpaul* (Fig. 3C and Table S1). Furthermore, we performed conventional PCR for the amplification of the *E. coli, M. abscessus*, *M. gordonae, Sal. typhimurium, Sal. newport, and Sal. saintpaul* DNAs in order to check for the removal of PCR inhibitors from the DNA extracted with the DTS assay. We observed that the genes from those bacteria were strongly amplified using extracted DNAs from both assays (Fig. 4A). This demonstrated that the DTS assay can be used to successfully extract DNA from six bacterial samples with good quantity and purity for use in downstream analysis (Table 1 and Table S1). Taken together, these characteristics make the DTS procedure an improved method for the extraction of DNA. The DTS procedure allows DNA to be captured through strong interactions with DMA immobilized on a thin film and does not require centrifuges or complicated fabrication steps such as deposition and etching.

Next, we evaluated the capacity of the DTS assay (30 min processing) compared to the Qiagen kit (60 min processing) with serially diluted eukaryotic cells. The sample solutions containing MCF7 cells ranging from 1 × 10^3^ to 10^5^ cells were in a same volume of 100 μL. As provided in Fig. 3B, we showed that the quantity and purity of the DNA extracted with the DTS assay is similar to that obtained by the Qiagen kit (Table 1). We also evaluated the capacity of the DTS assay with other eukaryotic cells including NCI-H1975 (lung tissue), CaCo-2 (colon tissue), T24 (bladder tissue), U937 (lymphocyte), and Jurkat (peripheral blood) (Fig. 3D and Table S1). Furthermore, in order to check the sufficient removal of PCR inhibitors in the DNAs extracted from the DTS assay for use in downstream analysis, we performed both genetic and epigenetic analyses of several biomarkers from the DNA extracted from both assays (Fig. 4B,C). For the genetic analysis of the HRAS gene, which is a DNA biomarker of bladder cancer^48^,^49^, we used RT-PCR with in-house-designed amplification primers (Table S2). We observed that the *HRAS* gene from MCF7 cells was strongly amplified using the DNA extracted from both assays (Fig. 4B). Furthermore, we performed methylation specific endonuclease digestion for the epigenetic analysis of the *RARβ* (a common DNA methylation biomarker in several cancers)^50^,^51^,^52^ by using the extracted DNA (Fig. 4C). In the methylation analysis, the quality and quantity of the DNA extracted are of crucial importance due to the low proportion of methylated DNA in genomic DNA pools^53^,^54^,^55^. For the methylation specific endonuclease digestion, the DNA extracted using both assays was digested by *MspI* and *HpaII*, which can recognize the methylation sites at CCGG sequences and cleave those sequences. The gel electrophoresis results after PCR showed that the methylated *RARβ* genes from both assays are amplified after HpaII digestion, and the product represents the existence of the methylated region (Fig. 4C). On the other hand, *MspI* was used as a negative control as, unlike *HpaI*, it can cleave sequence methylated at the internal C in the CCGG sequence, and this did not result in amplicon production as fragmentation of the amplified region of the gene had occurred. We showed that the DNA extracted from the DTS assay was sufficient to detected epigenetic variants of this biomarker gene (Fig. 4C). Taken together, the DTS assay can be useful for the extraction of DNA from cancer cells in sufficient quantity and purity to allow subsequent downstream analysis such as the genetic and epigenetic analyses of biomarkers.

### Validation of DTS assay on body fluids

**Figure 5:**
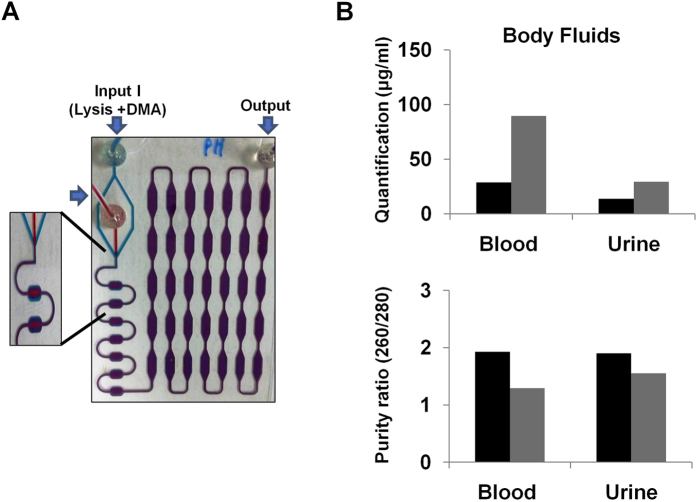
Utility of the DTS assay with human body fluids (whole blood and urine). (**A**) The DTS assay with a microfluidic system could be used to extract DNA from body fluids such as whole blood and urine within 30 min. (**B**) The colors represent the type of the assay for the extraction of DNA; Black (Qiagen kit), and Grey (DTS).

Finally, to determine whether the DTS assay could be used to extract DNA from body fluids such as whole blood and urine within 30 min, the human body fluids (whole blood or urine) were injected seperately with the mixture solution (DMA and lysis buffer) by using two inlets (Inlet I; lysis buffer and DMA, Inlet II; sample) and one outlet (waste/collection). Inlet II used for the mixture solution was divided into two streams that sandwich the sample from Inlet I (Fig. 5A). We compared the DTS assay with the Qiagen kit to validate the efficiency of the assay on human whole blood or urine samples. First, either 200 μl of whole blood or urine was used for the DNA extraction with both assays. To obtain the extracted DNA using the DTS assay, we followed a three-step process: (1) injecting the samples and lysis buffer with DMA separately into the device, (2) incubation at 56 °C for 20 min, (3) wash and elution. Figure 5B shows that the quantity and purity of the DNA extracted from the DTS assay were similar or better than those from the Qiagen kit (Table 1). As our expectation, highly purified DNA was also obtained by using the DTS assay with both whole blood and urine samples. Therefore, as shown here, the DTS assay represents a simple, useful, and rapid process for the extraction of large amounts of high-quality DNA from human body fluids.

## Discussion

Here, we report the utility of a DTS assay that couples DMA (a non-chaotropic reagent) and thin film technology to efficiently extract DNA from various complex samples (eukaryotic cells, bacterial cells, and human body fluids). The DTS assay shows several advantages, overcomes the limitations of other extraction techniques, and offers simplicity of use (single step), rapidity, lower costs and labor, as well as allows the extraction of a high quantity and pure DNA (Table S3). Furthermore, the DTS assay does not requiring centrifuges or complicate fabrication (Table S3). Thus, the DTS assay should become a useful and common laboratory tool for the extraction of DNA in the field of life sciences and could be applicable as a point-of-care (POC) detection system by integration with an isothermal solid phase amplification/detection (ISAD)^56^ that has been recently developed by our group for the clinical application. Going forward, we envision that the DTS assay will potentially facilitate increased research quality in the fields of life sciences and biomedical engineering by reducing the researcher’s hand-on time and use of large instruments.

## Methods

### Microfluidic chip development

A thin film device for DTS assay was composed of a microfluidic chamber for DTS assay (Fig. 2A). The microfluidic chamber consisted of several slot-type microwells connected to each other with a flow path in the chamber to extract of DNA from sources. The device was simply and quickly fabricated by a laser cutting machine (Universal Laser Systems, Scottsdale, USA). First, in order to fabricate the microfluidic chamber, the laser cutting machine cut the design of the microfluidic chamber into a 300 μm thick double-sided tape (a 100 μm thick polyester film, sandwiched between the 100 μm thick double-sided tapes) (Fig. 2A). Second, the thin film (upper and lower) was cut to the same dimensions as the microfluidic chamber using the laser cutting machine. Through holes were fabricated in the upper thin film. Each laser-cut film was attached to the top and bottom permanent adhesive surfaces of the laser-cut microfluidic chamber to generate functionality. As a result, the chamber height was approximately 300 μm and the total volume was 300 μL (Fig. 2A). Third, in order to fabricate tubing adapters for sample flow, a cast acrylic sheet (MARGA CIPTA, Indonesia) with 3 mm thickness, attached to the double-sided tape on one side, was cut and drilled by the laser cutting machine. The fabricated adapters were attached to the inlet and outlet of the microfluidic chamber. Then, pre-cut Tygon tubing (AAC02548; Cole-Parmer, Vernon Hills, USA) was placed in the hole of the adapter and sealed using epoxy (Fig. 2A). Finally, to use the DTS device as a DNA extraction assay, the modified protocol was used. In order to create amine group of the thin film surface, the surface were first treated with oxygen plasma for 10 min and immersed in a solution of 2% 3-aminopropyltriethoxysilane (APTES, Sigma-Aldrich) in H_2_O solution for 10 to 60 min at 65 °C, followed by thorough rinsing with DI (de-ionized) water. To cure the surface, they were dried under a nitrogen stream quickly. Water contact angle measurements of the amine-modified surface demonstrated that the hydrophilicities of the surfaces were changed significantly depending on temperature and incubation time using Drop Shape Analyzer, DSA100 (KRUSS, Germany). After silanization with APTES on the surface for 10 min at 65 °C, the surface hydrophilicity was increased (ca. 30–40 °C). At this time, the DTS device was ready for extraction of DNA from the various sources. Store device at room temperature until use.

### DTS assay operation

To extract DNA using the DTS assay [300 μL volume, 8.4 cm × 3.7 cm], we prepared the assay solution optimized for DNA extraction. For optimized reaction, lysis buffer containing 100 mM Tris-HCl (pH 8.0), 10 mM EDTA, 1% SDS, and 10% Triton X-100 was mixed with DMA (50 mg/mL). To start the assay, 100 μL of sample from cells, bacteria, blood, or urine were mixed with the 200 μL of the assay solution. The DTS device was then placed on either an incubator or a thermoelectric cooler (TEC) with controller (Alpha Omega Instruments) to keep a constant temperature (56 °C) for 20 min in order to extract DNA from the sources and capture DNA through DMA reagent on the surface. Following washing step with PBS to get rid of debris from the samples, the Elution buffer (10 mM sodium bicarbonate, pH 10.6) used to collect the DNA extracted within few minutes. The qunatity and purity of DNA extracted was measured determining the ratio of the optical densities of the samples at 260 nm (DNA) and 280 nm (protein) using Enspire Multimode Plate Reader (PerkinElmer). For comparison of the DTS assay with a conventional DNA extraction method, QIAmp DNA mini kit was used according to the manufacturer’s protocol (Qiagen, Hilden, Germany).

### Eukaryotic cells and bacterial cells

Six eukaryotic cells [MCF-7 (breast), NCI-H1975 (lung), CaCo-2 (colon), T24 (bladder), U937 (lymphocyte), and Jurkat (peripheral blood)] were maintained in plastic culture dishes with high-glucose Dulbecco’s ModifiedEagle’s Medium (DMEM, Life Technology) supplemented with 10% fetal calf serum (FCS) in a 37 °C humid incubator with 5% ambient CO_2_. After culture of the eukaryotic cells, the genomic DNA was then extracted from the cells using proteinase K and QIAmp DNA mini kit (Hilden, Germany). End-point PCR and Real Time (RT)-PCR were performed to check the quantity and purity of DNA for downstream analysis. The forward and reverse primers of several genes (*HRAS, Actin, and RARβ*) were synthesized at the usual length of around 24 bp (Table S2). The end-point PCR process consisted of an initial denaturation step at 95 °C for 15 min; 45 cycles of 95 °C for 45 s, 59 °C (*RARβ*) for 45 s, and 72 °C for 45 s; and a final elongation step at 72 °C for 10 min. 5–10 μL of DNA were amplified in a total volume of 25 μL containing 1× PCR buffer (Qiagen), 2.5 mM MgCl_2_, 0.25 mM deoxynucleotide triphosphate, 25 pmol of each primer, and 1 unit of Taq DNA polymerase (Qiagen). For RT-PCR process, the following procedure is modified from LightCycler 2.0 Instrument protocol (Roche Diagnostics). 5–10 μL of DNA were amplified in a total volume of 20 μL containing 4 μL of LightCycler FastStart DNA Master mix, 25 pmol of each primer, and 2 μL of 1× PCR buffer (Qiagen), 2.5 mM MgCl_2_, 0.25 mM deoxynucleotide triphosphate, 25 pmol of each primer, and DI water. An initial pre-incubation cycle of 95 °C for 10 min was followed by 50 cycles of 95 °C for 10 s, 58 °C (*HRAS and Actin*) for 30 s, and 72 °C for 10 s, and by cooling step of 40 °C for 30 s. The amplified products with SYBR Green signals were carried out on a LightCycler 2.0 (Roche Diagnostics). Next, to examine the epigenetic variant of *RARβ* from the DNA extracted, the DNA was mixed with either *MspI* or *HpaII* solution (150 μL) to digest the DNA at 37 °C for 20 min in a single reaction tube. After the digestion step, the tube was placed at 80 °C for 10 min for inactivation of the restriction enzymes. Following the inactivation step, the digested DNA used as a template for epigenetic analysis of *RARβ* gene obtained from both assays by using conventional PCR.

Next, in order to elucidate the capacity of DTS assay with bacterial cells, we performed PCR-based DNA amplification by using the extracted DNA using the DTS assay (Fig. 2). All primers used for conventional PCR of *E. coli, M. abscessus, M. gordonae, and three Salmonella strains* are described in Table S2. For optimized reaction, lysis buffer containing 100 mM Tris-HCl (pH 8.0), 10 mM EDTA, 1% SDS, and 10% Triton X-100, and 20 mg/mL of Lysozyme was mixed with DMA (50 mg/mL). Conventional PCR was performed to verify the efficiency of the proposed technique for the genetic analysis. *E. coli* XL1 Blue strain was inoculated in Luria-Bertani (LB) medium with 50 μg/ml tetracycline and incubated overnight at 37 °C under shaking condition. Samples ranging from 10^3^ to 10^7^ CFU (colony formation unit) were used for the study. Bacterial DNA was extracted from *E. coli, M. abscessus, M. gordonae, Sal. typhimurium, Sal. newport, and Sal. saintpaul* cultures by using both the DTS and Qiagen assays. For genetic analysis of the bacteria genes, 2 μl of the DNA extracted from each assay such as the DTS and Qiagen was amplified in a total volume of 25 μl containing 1× PCR buffer (Qiagen, Hilden, Germany), 2.5 mM MgCl2, 0.25 mM deoxynucleotide triphosphate, 25 pmol of each primers, and 1 unit of Taq DNA polymerase (Qiagen, Hilden, Germany) at 95 °C for 15 min; 45 cycles of 95 °C for 30 s, 60 °C (*M. abscesuss, M. gordonae and Sal. strains*) for 30 s, and 72 °C for 30 s; and a final elongation step at 72 °C for 7 min. PCR amplicons were visualized by gel electrophoresis, which was used to separate PCR products on a 2% agarose gel containing ethidium bromide (EtBr) (Sigma-Aldrich). The gel was visualized using a Gel Doc System (Bio-Rad). Determination of DNA concentration and purity was done by UV spectrophotometer (Perkin-Elmer) (Table 1 and Table S1).

### Body fluids with DTS assay

To validate the ability of the DTS assay with human body fluids, the 200 μL of samples (whole blood and urine) was injected for DNA extraction. Blood and urine samples from one healthy donor were obtained on protocols approved by the Institutional Review Board of NUHS (National University Health System), Singapore. Institutional approval and informed consent from the healthy human were obtained in writing. All experiments were performed in accordance with relevant guidelines and regulations. The DTS assay with the microfluidic channel that consisted of two inlets (I; lysis buffer and DMA, II; sample) and one outlet (waste/collection). The buffer solution is divided into two streams that sandwich the sample within the microfluidic channel. All samples and reagents are sequentially delivered to the microchip as follows Fig. 5A; Inlet I: buffer solution containing lysis buffer with proteinase K and DMA – injecting the solution into the two lines of microchannel to be used as a lysis buffer; Inlet II: sample – injecting the sample into the microchannel; Outlet: washing and elution – injecting the wash buffers (PBS) to purify the sample and eluting the DNA from the surface of the thin film. When using the DTS assay, both the sample and buffer solution were injected with a syringe pump (KD Scientific, MA) into Inlet I and II at a flow rate 1.5 ml/hr for 10 min. Then, the cartridge was incubated at 56 °C for 20 min to extract and purify the DNA from cells. PBS buffer by syringe pumps was added to Inlet II at a flow rate of 4 mL/hr for 10 min. Finally, the extracted DNA was eluted with elution buffer at volume of 100 μL. In addition, 200 μl of whole blood or urine was used for genomic DNA extraction as reference material using a QIAmp DNA mini kit (Hilden, Germany). All extracted DNA was determined the concentration and purity of DNA by UV spectrophotometer (Perkin-Elmer) (Fig. 5B and Table 1).

## Additional Information

**How to cite this article**: Shin, Y. *et al.* Dimethyl adipimidate/T hin film Sample processing (DTS); A simple, low-cost, and versatile nucleic acid extraction assay for downstream analysis. *Sci. Rep.* **5**, 14127; doi: 10.1038/srep14127 (2015).

## Supplementary Material

Supplementary Information

## Figures and Tables

**Table 1:** Comparisons of DTS assay and Qiagen kit using several samples.

	Types	Conc.	Current (DTS)		Conventional (Qiagen kit)	
			Quantity (μg/ml)	Purity (260/280)	Quantity (μg/ml)	Purity (260/280)
**Samples**	Eukaryotic Cell (MCF7)	10^7^ cells (RT)	30.17 ± 7.64	1.59 ± 0.17	No recommends	
		10^7^ cells (56 °C)	54.31 ± 22.2	1.86 ± 0.18	35.63 ± 7.96	1.68 ± 0.21
		10^5^ cells	39.14 ± 7.98	1.66 ± 0.03	38.56 ± 1.84	1.82 ± 0.01
		10^4^ cells	6.49 ± 1.31	1.86 ± 0.05	10.34 ± 1.31	1.67 ± 0.07
		10^3^ cells	4.14 ± 0.37	1.63 ± 0.02	5.80 ± 0.95	1.69 ± 0.03
	Bacterial Cell (*E. coli*)	10^7^ CFU	10.34 ± 3.38	1.69 ± 0.05	7.87 ± 0.06	1.59 ± 0.01
		10^5^ CFU	12.04 ± 1.98	1.72 ± 0.04	4.74 ± 0.88	1.57 ± 0.05
		10^3^ CFU	10.27 ± 1.86	1.75 ± 0.14	5.39 ± 1.30	1.58 ± 10.5
	Body Fluids	Blood (200 μl)	89.65 ± 53.6	1.59 ± 0.09	28.79 ± 1.9	1.93 ± 0.11
		Urine (200 μl)	29.31 ± 2.44	1.55 ± 0.07	13.79 ± 5.97	1.90 ± 0.96
