# Supplementary material for: Dimethyl adipimidate/Thin film Sample processing (DTS); A simple, low-cost, and versatile nucleic acid extraction assay for downstream analysis
Source: Sci Rep. 2015 Sep 15;5:14127. doi: 10.1038/srep14127 (PMC4569962; doi:10.1038/srep14127)
Supplement: Supplementary Information [file srep14127-s1.doc]

**Dimethyl adipimidate/Thin film Sample processing (DTS); A simple, low-cost, and versatile nucleic acid extraction assay for downstream analysis**

Yong Shin^1,*^, Swee Yin Lim^1^, Tae Yoon Lee^1^, [Mi Kyoung](http://ime-intraspsvr/photoboard/BE  Biophotonics/Park Mi Kyoung.jpg) Park^1^

^1^Institute of Microelectronics, A*STAR (Agency for Science, Technology and Research), 11 Science Park Road, Singapore Science Park II, Singapore117685.

^*^Correspondence and requests for materials should be addressed to Y.S. (email: [shiny@ime.a-star.edu.sg](mailto:shiny@ime.a-star.edu.sg))

Supporting Information:

Methods

Capability of DMA using Silicon microring resonators (SMRs)

To monitor the capability of DMA binding with either DNA or protein (MBD4; methyl domain binding protein 4), we used the silicon microring resonator (SMR) as a biophotonic based detection device. The structure and fabrication of the silicon microring resonators have been previously described^38-39^. For amine modification on the surface of the SMRs, we modified the surface modification protocol for the SMR as previously described^38-39^. Briefly, the sensors were treated with oxygen plasma and immersed in a solution of 2% 3-aminopropyltriethoxysilane (APTES) in a mixture of ethanol-H2O (95%: 5%, v/v) for 2 h, followed by thorough rinsing with ethanol and DI water. To cure the sensors, they were dried under a nitrogen stream and heated to 120 °C for 15 min. After modification, an acrylic chamber was bonded onto the sensor to enclose the microring sensor area for optical measurements. For obtaining the baseline by optical wavelength measurement under static conditions (no flow), we applied 16 μl of PBS solution to the chamber. During the reaction (up to 10 min), we did not note any significant baseline shift. Then, a mixture of 10 μl of DMA agent (25 mg mL−1; Sigma-Aldrich) with either 10 μl of human genomic DNA (1 μg) or 10 μl of MBD4 protein (1 μg; Abcam) was added to the chamber at room temperature by using a micro-pipette after removing the PBS solution. To monitor the binding capability of DMA/DNA or DMA/protein complex to the amine-modified surface, the wavelength shift was collected every 5 min, up to 20 min after the addition. In order to eliminate unbound molecules or excessive mixture of DMA and DNA or protein, the surface of the sensor was washed twice with PBS buffer. The wavelength shift was also collected every 2.5 min, up to 10 min during the washing step. Finally, 50 μl of the elution buffer (10 mM sodium bicarbonate, pH 10.6) was used for elution of the captured DMA and DNA or protein with monitoring of the wavelength shift every 5 min, up to 10 min (Fig. S1B).

***
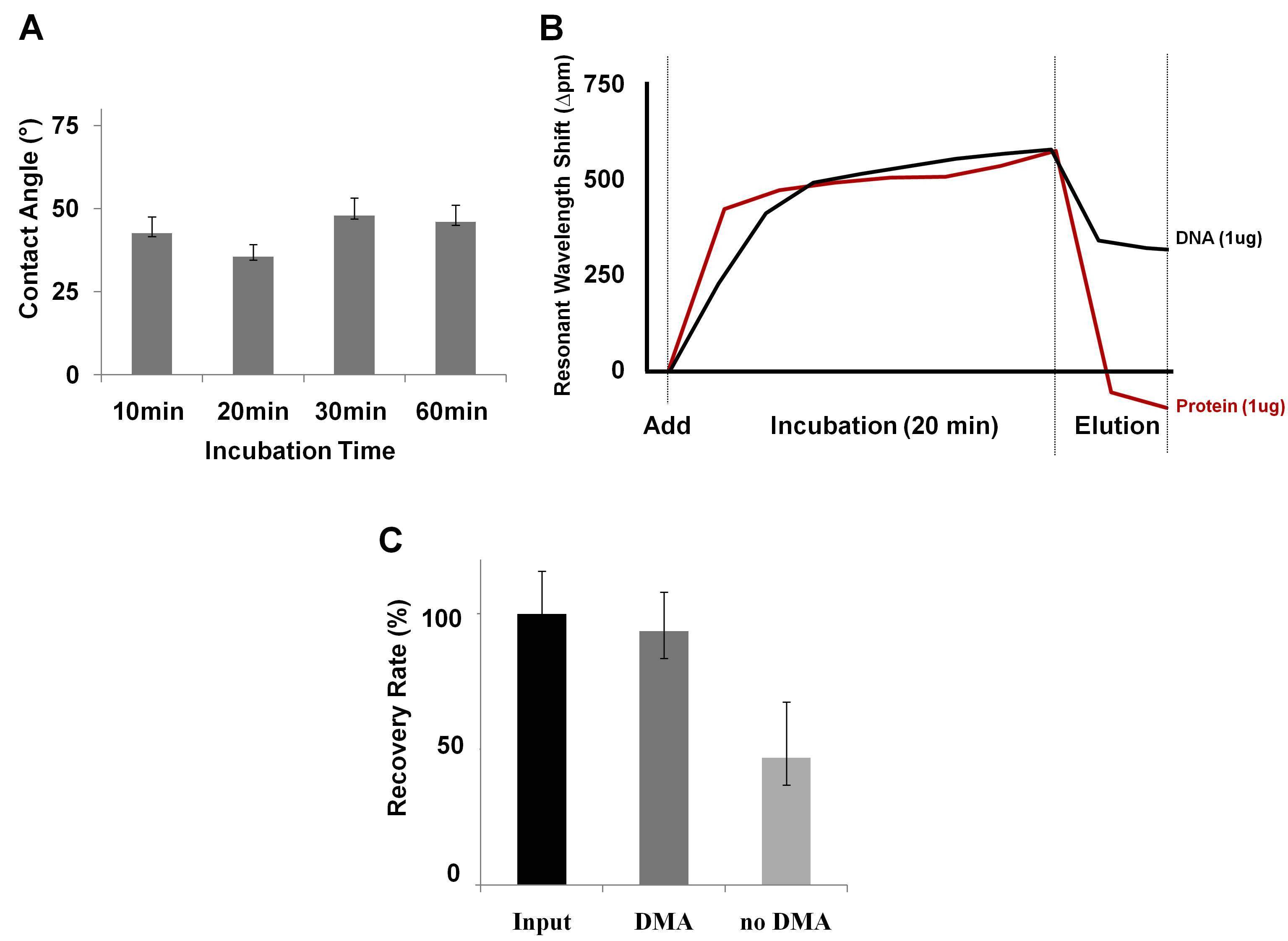
***

***Figure. S1.*** Fundamental characterization of DTS assay. (A) Hydrophilicty of the surface by the amine group was rapidly changed within 10 min at 56 ºC. All error bars indicate the standard error of the mean based on at least 3 independent experiments. (B) Capability of DMA using silicon microring resonators (SMRs). Resonance wavelength shift shows the results of DMA interacts with either DNA (black) or Protein (red) for 20 min incubation. Solution including DMA with either DNA (human gDNA) or protein (MBD4) was pre-mixed and added into the silicon sensor. The solution was then incubated at RT for 20 min to cross-link the molecules on the surface of the sensor. PBS buffer was used for washing the unbounded molecules. The DNA captured was isolated by the elution buffer. The reaction was monitored by the measuring of the wavelength shift on SMRs. (C) Recovery rate of the input DNA (1μg) with and without DMA.


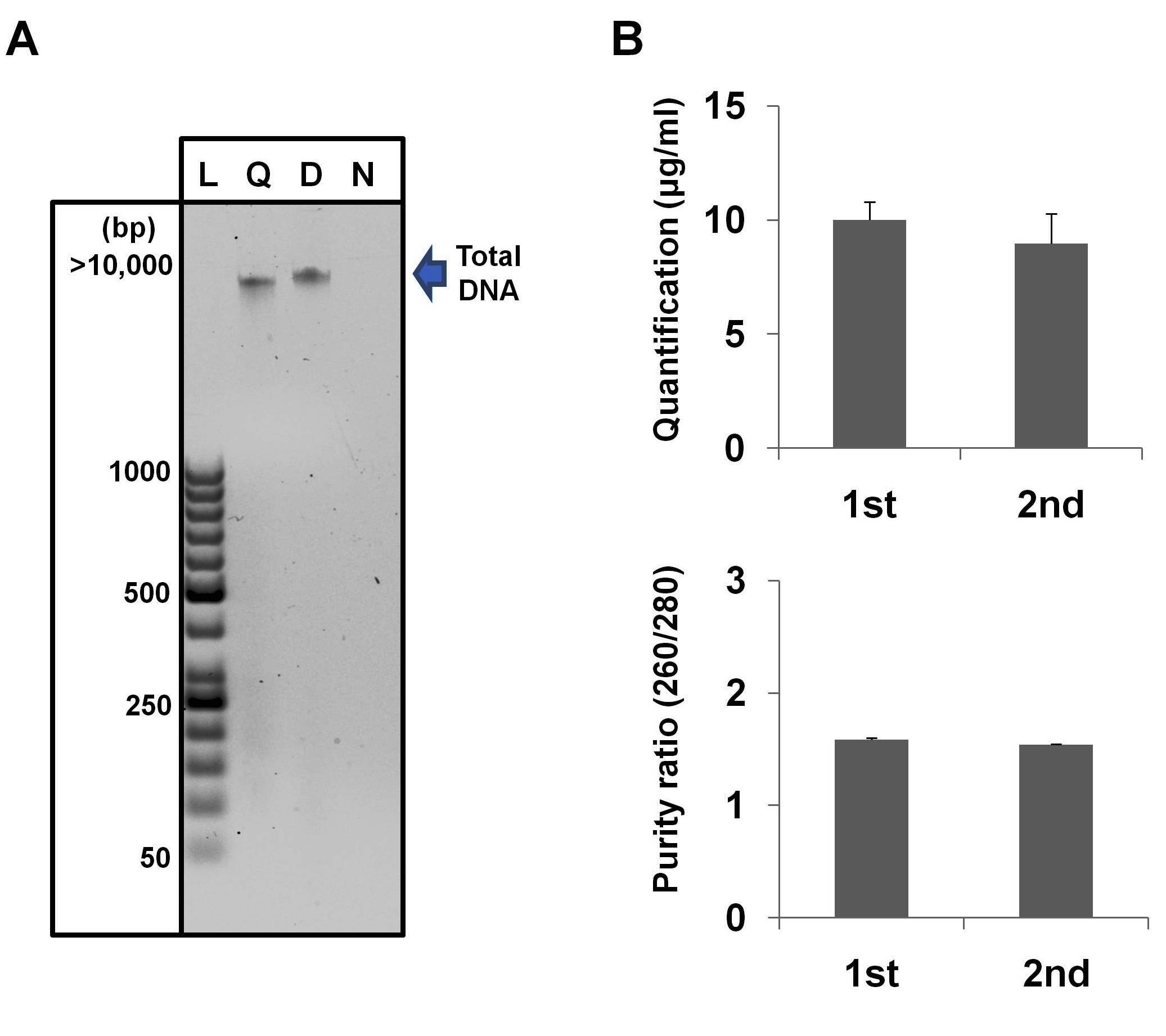


***Figure. S2***. (A) Integrity of total DNA extracted from eukaryotic cell using DTS assay. The gel electrophoresis analysis on 1% agarose gel for 30 min with 5ul of total DNAs from both Qiagen kit and DTS assay. [L: 50 bp DNA ladder, Q: DNA from Qiagen kit, D: DNA from DTS assay, and N: no DNA]. (B) Reuse testing of DTS device. The quantity (upper) and purity (lower) of the DNA extracted from the eukaryotic cells (NCI-H1975) with 1^st^ and 2^nd^ experiments in a single DTS device. All error bars indicate standard error of the mean based on at least 3 independent experiments.

*Table. S1. Quantity and purity of DNA extracted from DTS using several samples*

|  |  | | **DTS assay** | | |
| --- | --- | --- | --- | --- | --- |
|  | **Types** | | **Quantity (μg/ml)** | **Purity**  **(260/280)** | **qRT-PCR**  **(ct value)** |
| **Samples** | Eukaryotic Cells  (10^5^ cells) | NCI-H1975 | 13.10 ± 2.94 | 1.64 ± 0.05 | 25.36 ± 0.19 |
|  |  | CaCo-2 | 43.27 ± 12.41 | 1.68 ± 0.06 | 24.51 ± 0.98 |
|  |  | T24 | 23.96 ± 5.18 | 1.68 ± 0.07 | 22.46 ± 0.47 |
|  |  | U937 | 23.62 ± 2.33 | 1.77 ± 0.01 | 24.05 ± 0.22 |
|  |  | Jurkat | 11.03 ± 0.79 | 1.63 ± 0.007 | 25.34 ± 0.99 |
|  | Bacterial Cells  (10^7^ CFU) | *M.abscesuss* | 6.03 ± 0.73 | 1.65 ± 0.003 | Figure 4A |
|  |  | *M.gordonae* | 8.44 ± 2.19 | 1.60 ± 0.002 |  |
|  |  | *Sal. Typhimurium* | 18.44 ± 3.39 | 1.69 ± 0.09 |  |
|  |  | *Sal. Newport* | 13.10 ± 0.92 | 1.59 ± 0.03 |  |
|  |  | *Sal. Saintpaul* | 13.79 ± 8.51 | 1.61 ± 0.04 |  |

*Table. S2. Primer sequences for PCR/qPCR*

*Table. S3. Comparisons of requirements for using DTS assay and Qiagen kit*

| **Target Gene** | **Name** | **Primer Target** | **Sequence** |
| --- | --- | --- | --- |
| *Actin*  *(170bp)* | Forward Primer | *Actin (Exon)* | 5´- ATGGTGGGCATGGGTCAGA-3´ |
|  | Reverse Primer |  | 5´- GCCACACGCAGCTCATTG-3´ |
| *HRAS*  *(130bp)* | Forward Primer | *HRAS (Exon)* | 5´- TGGTGGTGGGCGCCGGCG-3´ |
|  | Reverse Primer |  | 5´- CCTATCCTGGCTGTGTCCTG-3´ |
| *RARβ*  *(184 bp)* | Forward Primer | *RARβ ( promoter)* | 5´- ATTTGAAGGTTAGCAGCCCG-3´ |
|  | Reverse Primer |  | 5´- GCATCCCAGTCCTCAAACAG-3´ |
| *E.coli*  *(138 bp)* | Forward Primer | *E.coli* O157:H7 | 5´- CAACTCTGGCTCCGTCTCTG-3´ |
|  | Reverse Primer |  | 5´- CATCATGCAAGCGGCCTCTG-3´ |
| *M.abscesuss*  *(440 bp)* | Forward Primer | *16S rRNA* | 5´- ACCACACACTTCATGGTGAGTGG-3´ |
|  | Reverse Primer |  | 5´- GCCCGTATCGCCCGCACGCACAC-3´ |
| *M. gordonae*  *(220 bp)* | Forward Primer | *16S Internal transcribed spacer* | 5´- AAGACCGGGTGCACGACAACAA-3´ |
|  | Reverse Primer |  | 5´- TGCGCCCTTAGACACTTACAAAC-3´ |
| *Salmonella*  *(116 bp)* | Forward Primer | *Salmonella pathogenicity*  *islands (SPl1)* | 5´- CAACGTTTCCTGCGGTACTGT-3´ |
|  | Reverse Primer |  | 5´- CCCGAACGTGGCGATAATT-3´ |

|  | **Types** | **Current**  **(DTS assay)** | **Conventional**  **(Qiagen kit)** |
| --- | --- | --- | --- |
| **Requires** | Main technique | DMA (no membrane)/thin film  Non-chaotropic reagent | Filther/membrane  Chaotropic reagent |
|  | Method steps | Less hands-on time  1. Lysis at 56 °C  2. Binding with DMA  3. Single Wash  4. Elution | Much hands-on time  1. Lysis at 56 °C  2. Binding to filter  3. Several Washs  4. Elution |
|  | Instruments | No centrifuge | Centrifuges |
|  | Processing time | < 30 min | < 60 min |
|  | Cost | Less $1 (per chip) | $ 7-8 (per column) |
